# Rachitis

**Published:** 1878-10

**Authors:** Joseph Fowler


BUFFALO

### Medical and Surgical Journal.

VOL. XVIII. OCTOBER, 1878. NO. 3.

### Original Communications.

——:o:——

*ART. I.—Rachitis.*—By JOSEPH FOWLER, M. D. Read before the Buffalo Medical Association, May 31st, 1878.

With few exceptions American writers upon the subject of this paper discuss it very briefly, probably for the want of personal experience—the disease prevailing to such a limited extent on this continent, and simply give their readers the result of foreign research and observation, perhaps indulging in a variety of hypothetical reasoning.

Rachitis is essentially a disease of infancy and childhood, and this may be the proper explanation of its omission in many of our principal Text-books of Practice; and even authors of works treating on infantile diseases sometimes fail to call attention to the affection, possibly because it is classified by almost unanimous consent, with bone diseases, and left for writers upon that subject to describe.

American literature, then, we find strangely deficient, only a few lengthy articles having been written upon the subject, of which probably the most exhaustive is that of Dr. Parry, which appeared in two numbers of the *American Journal of the Medical Sciences*, for 1872. It reviews all known opinions upon the subject, and gives his own personal experience in Philadelphia.

Most of the serious diseases of childhood, and I might say adult life, prevailing in one civilized country are usually found to exist in all others in a singularly relative proportion, as diphtheria, phthisis, the eruptive fevers, &c., and it strikes us as quite remarkable that in London a form of disease, possessing all the characteristics that rachitis does, should exceed in frequency all other infantile disorders, and still its existence be so very rare on the Western continent.

The first comprehensive description given of rachitis was by Glisson, and his associates, a little more than two hundred years ago, but this was ante-dated by a book written by David Whistler in 1645, from which Glisson acknowledges to have obtained the term *rickets,* and from the opinions of these gentlemen it was assumed or believed that the disease had its origin in Western England about this time, and from this and probably its constant appearance subsequently, it was known to the continental physicians as the English Malady, although some accounts given in ancient history of certain peculiar deformities of the human skeleton, it is not unlikely that the disease existed on the European continent long before Whistler and Glisson wrote about it.

Rickets, says Bristow, is a disease of early childhood, characterized by softening of the bones, with enlargement of the joint ends of long bones, enlargement of spleen and liver, and coincident symptoms of ill-health.

Erichsen describes it is "a disease of early life." In it the structure of the bones is changed, the earthy matter being deficient so that the bone continues to be soft, flexible and cartilaginous in structure, &c.

But few writers define the term so briefly, and all unite in a general description of the affection as just quoted.

The ætiology of rachitis does not seem to be well understood, notwithstanding the fact that the disease has been probably as closely studied as any other single affection.

Some would say it had its origin in the nutritive functions; others that the primary lesion was to be found in the osseous system. Again, this one speaks of it as a distinct disease, possessing characteristics peculiar to itself; and that one regards it as only a variety of a species in which might be included fragiletas osseum, osteo sarcosis, &c. Another makes brief mention of it under the head of scrofulous disease of bones.

To the unqualified statements of American writers, that rachitis is exceedingly rare on this continent, Dr. Parry takes exceptions, based upon his own observation, and says that in the children's department of the Philadelphia Hospital at least 28 per cent. of all the sick children between the ages of one month and five years that have come under his observation during the last three years, have been rachitic. He still goes on to say that he is irresistibly forced to the conclusion that rachitis is scarcely less frequent in Philadelphia than it is in the large cities in Great Britain and the continent of Europe.

The want of harmony between this statement and those of other American writers, where each has nearly the same opportunity for observing, naturally leads us to enquire into the causes of these differences of opinion. To prove the extent of the prevalence of rachitis on the European continent, Dr. Parry reports Rettershaine as having seen the disease in 31 per cent. of children under five years of age who applied for relief at the Medical Clinic at Prague. Gee found 30-3.10 per cent. of those under two years of age admitted to the Hospital for sick children in London. Merei stated that 20 per cent. of children in the upper classes were rachitic. Ritchie of Manchester says that out of 728 children under five years of age about 30 per cent. were afflicted with the disease.

Henoch confirms Rittershaine's estimate. I mention this for the reason that Dr. Parry says they are all the figures which we possess upon the subject. Bauer, in his work on diseases of bone, says, when speaking on the subject of rachitis, that during a practice of fifteen years he had not seen an aggravated ease. Markoe says the disease is so rarely seen in this country that he cannot speak from his own observation. Meigs and Pepper regard the disease as vastly more common among the poorer classes in London than among the same classes in this country.

To my knowledge no statistics on this point have been published in this country, aside from those mentioned by Dr. Parry in his paper on this subject. I have attempted to gather some additional figures, but find most of the Hospital and Dispensary reports almost valueless, not containing a classified or tabulated statement of their work.

At the Columbia Hospital Dispensary, Washington, D. C., from Sept. 1st, 1869, to July 1st, 1872, 1,028 children were presented for treatment and their diseases classified, out of which eleven (11) were found to have rachitis.

In the absence of more important facts, I find in the report of the Buffalo Dispensary Association, that from Oct. 1st, 1876, to the present time, fully 500 children came under my observation, and of this number not a single well-marked case of rachitis was observed. During the previous year, by reference to the printed report, 369 children were treated, not one of which had the disease. For one and a half years' service as Resident before this time I do not recollect of seeing a single case of rachitis.

I am informed by the resident physician in charge of the Erie County Alms-house during the years 1864 5, that fully 600 sick children were presented for medical treatment, and of this number but one or two cases of rachitis were seen.

Dr. Parry says, " he can only account for this difference of opinion between American writers and himself, that while they recognize the grave forms of the disease, they fail to appreciate the characteristic symptoms of the early stages of the affection."

Now, if we can see what the so-called characteristic symptoms are upon which Dr. Parry bases his diagnoses in the early stages, before there is any manifest change in the osseous system, perhaps we can discover an explanation for the frequent occurrence of the disease in his own private and hospital practice, as compared with that of others in his own city and elsewhere. Again, when he says the disease has no definite course to run, and one that may be arrested at any stage of its progress; nearly always preventable, and if treated properly will nearly always end in recovery, we might go back still further and ascertain what the gentleman terms rachitis.

In his essay he points out three important symptoms, two of which are almost invariably present during the stage of incubation, so-called by *Guersant,* and almost pathognomonic of the disease. These, he says, are sufficiently striking and should always lead the physician to suspect the presence of this cachexia. Nay, more; they are so characteristic of the affection which we are discussing, that it at once becomes the duty of the medical attendant to place the patient upon treatment for rickets.

Making this statement so strong it is proper to assume that upon the presence of these he fixes his diagnoses, whether subsequent bone changes take place or not.

The two symptoms just alluded to are profuse perspiration of a local character, and heat of surface, most marked at night time. The third, tenderness of the surface, pointed out by Jenner, Meigs and Pepper, Neimeyer and others as either preceding or accompanying the profuse sweating and heat of surface, as Dr. Parry thinks, is very often wanting during this stage, and when present is associated with the bone leisions of the second stage or stage of deformation. Markoe says the desire to be cool at night is so common among children otherwise perfectly well that it cannot of itself be considered characteristic, but thinks that the most characteristic symptom of all is a general tenderness to the touch of the whole body.

In addition to these, Dr. Parry goes on to state, are other symptoms of minor importance, which are irregularly associated with those of the first stage, such as enlargement of the veins of the scalp, more or less emaciation, &c. The three symptoms considered to be of so much importance were pointed out by the old writers, but Dr. Parry "thinks they have not received the attention of the profession which their importance demands."

The sweating spoken of takes place usually during sleep and under the most trivial excitement or physical effort. The pillow upon which the child lies will be found saturated with perspiration where it comes in contact with its head. It rolls its head from one side to the other until its occiput is worn bald. Its restlessness appears unceasing. During all months of the season and all seasons of the year the child may be found at almost any hour of the night uncovered and naked.

Is not this the history of hundreds of children who remain stout and healthy through their infancy and childhood, and the presence of rachitis never enters the mind of the family physician.

The attention of the physician is often called to just this condition of cephalic sweating and nocturnal restlessness, with often increased cutaneous temperature, particularly of the former, as there seems to be a notion among mothers and nurses that this profuse perspiration is due to a largely developed brain, and we hear them say the child has too large a head, or is too smart to live; but, fortunately, our children do not often die from such causes. That there are definite predisposing causes no one will deny, but what they are we do not know.

For ourselves, on account of the rarity of the disease among us, no reasonable opportunities are offered for ascertaining in what the morbific element is contained or of what it consists.

It has been thought by some that syphilis might act as the great predisposing agent, but this theory lacks confirmation by foreign physicians; surely if the doctrine were tenable American practitioners would not want for rachitic patients, for syphilis is not by any means a scarce possession in this country. Heredity has been traced only to isolated cases.

Whether or not it is dependent on a scrofulous, tubercular or strumous diathesis, opinions are as diversified as they are numerous. In support of its scrofulous origin stand the names of Sir Charles Bell, Simon, Dupeytron, Watson, Whitehead, Condee, Hering, Rittershaine, Stewart, Merei, Aitken, Willshire and others, among whom are some American writers, while Meigs and Pepper, Holmes, Jenner, E. Smith, Routh, Markoe, Parry, and particularly the more recent writers on the subject, believe the disease is not due to hereditary influences unless in exceptional cases, and the cause must be looked for in the character of the surroundings of the child and its life-giving principles. There are others who believe it is sometimes due to one and sometimes to the other, or the combined influences of both.

Although many of the old writers believed the disease was as frequent in the upper classes as among those of the poor, the observations made during the last half century would hardly corroborate this opinion, or that of Bauer, who says rachitis is eminently a disease of pauperism, but would go far to establish an intimate relation between the causes of the affection and unwholesome nourishment, etc., in many cases. The theory that rachitis was due to the presence of bronchitis, whereby the blood was not thoroughly oxidized and carbonic acid allowed to accumulate in the system, which, according to Virchow, is the agent holding the elements of bone in solution before they are permanently deposited, re-dissolves them when they are carrried off as an excretory product, has not been favorably received. Among others, one of the most ancient theories ever advanced attributes the causation of the disease to a morbid state of the nervous system. Although much progress has been made in the study of nervous disorders, I am not aware of any recent discoveries and evidences in support of it.

If muscular activity developes the size and strength of the bones to which the muscles are attached in the same proportion as it does the muscles themselves, and inactivity of the latter produces atrophy of both, it would seem that an unhealthy condition of the bones might be induced by a perverted or deficient nervous power. With our present knowledge of the causes of rachitis, we know that it is most frequently found in illy-ventilated habitations where there is a cold, damp atmostphere, and among those children least cared for, subsisting upon unwholesome nourishment, and we might add, good food unsuited to the age of the child. In short, those influences which we believe to increase the fatality of scarlatina, rubeola, and develope malignant diphtheria, etc., are probably the same as favor the appearance of rachitis *more than all others.* This view I believe to harmonize with the opinions of recent observers who have watched carefully the developement and progress of the disease in the large cities of Europe.

To ascertain what influence improper nourishment would have, if any, in the developement of rickets. M. Guerin made an interesting experiment on young puppies while they were still dependent on their mother for nourishment. The natural food was discontinued and raw meat given instead, when it was found at the end of four or five months rachitis was well marked. This leads us to say that rickets has been seen in the wild and domestic animals, as well as in the human being. Of these can be mentioned the lion, monkey, horse, ox, pig, sheep, &c. Geese and ducks have been known to suffer from enlargement of the extremities of bones, with curvature of the long bones when confined in damp, cold apartments, but soon recover after being removed to dry, warm pens, and well nourished.

Rachitis appears in children between the ages of six months and two and a half years, rarely seen before or after these ages, a time in life which might justly be called the *critical period of infancy*, a time when the process of dentition is most active, and when important physiological changes are going on in the intestinal tract and elsewhere, when the function of nutrition is most susceptible of perversion.

Leaving the maximum age in children in which rachitis usually developes itself, passing on to adult life, we rarely observe any similar forms of disease attacking the bony skeleton. Again, leaving this period, and as we approach that of declining old age, occasionally have been seen adults suffering from extreme fragility and flexibility of the bones, which in many particulars seems identical to rachitis, but which has been classed by systemic-writers as a distinct disease of itself, on account of certain peculiarities it is said to possess, differing both in pathology and physical symptoms.

I allude to Osteo-Malacia, Osteo Sarcosis, Mollites Osseum.

In all the cases of this disease reported no two have been identified in their nature, and it is impossible to fix upon a definite train of symptoms which either precede or accompany the disease as it progresses.

Among those suffering from this disease many curious phenomena have been witnessed. Giants have been reduced in physical proportions to mere children in size.

The prophet *Getleb* was so flexible that he could be folded and packed in almost any imaginary manner.

* Osteo Sarcosis appears, says Bristowe, in the American reprint of his book on Theory and Practice, published two years ago, to have been recognized, indeed, only in women, and for the most part in women who have borne children. This gentleman must be in error in making this statement, or else is in ignorance of, or does not consider worthy of consideration the observation of *Killian*, who says it most commonly occurs in females but not invariably. *Litzman* collected 131 cases, of whom 83 were females in the parturient state, and of the remaining 46, 35 were females and 11 males. To these can be added other similar statistics which American authors mostly accept without comment.

By some this disease is believed to be only a variety of rickets attacking adults, and is so treated in the *Cyclopædia of Practical Medicine.* Efforts to ascertain the cause of Osteo Sarcosis have been just as unsatisfactory as those to determine the definite cause of rachitis, which at the present must be acknowledged as exceedingly obscure.

*Holmes* makes a distinction between the two diseases, and says rickets is an affection of early life, closely allied to scrofula in its causation and in its cure; it is peculiarly amenable to treatment; and under favorable circumstances the constitutional cachexia, which is its essence, readily disappears as the child grows. Mollites, he still adds, hardly ever makes its first appearance till after middle life; it shows no alliance with scrofula, and is not amenable either to the remedies for that disease, or as far as known, to any other remedies, but pursues its course unaffected for good by any medical treatment. He thinks malacosteon has a strong resemblance to malignant disease.

In the developing stage of rachitis there is said to be an entire absence of pain, while in Osteo-Sarcosis, pain is constant and severe. In rachitis there is a want of deposition of the earthy salts, while in malacosteon these same conditions exist with complete destruction, disorganization and resorption of the hardened bone, which cannot be restored. A rachitic bone is only modified in character, and susceptible of reorganization and restoration.

Of all the phenomena to be studied in an unmistakable case of rachitis, none are so important and interesting as the peculiar changes which take place in the osseous system. The bones not only become soft and pliable but undergo changes in form, the ends of the long bones enlarge mainly at the expense of their cartilaginous covering, and soften in consequence of deficient ossific deposit. The flat ones become thickened, more marked at their sutural margins than at their central points of ossification, while in some cases there is softening and resorption of the earthy matter at certain situations in the cranial bones, usually of the occipital and parietal. They are found at points which have been longest ossified, and probably due to pressure of the pillow upon one side, and the intracranial substance on the other. As many as 30 or more of these spots having been seen in the same individual at the same time.

Associated with this condition of craniotabes, it is not uncommon to find laryngismus stridulus, which, according to some writers is almost invariably present, and is only found in rachitic children. Whether this be so or not, to say the least, it is an unfavorable symptom, and those so affected usually die.

Accompanying these changes is an arrest of growth, which becomes most apparent in the face, giving the skull the appearance of enclosing a largely developed brain, but according to Dr. Shaw who has determined the relative size of rachitic skulls by actual measurement of many, says this is not the fact, but on the contrary they invariably fall short of the average size. This arrest of development preserves for a time the fœtal type, and the appearance is only imaginary and not real, and the prominent forehead and precocity of intellect delusive in character.

Dr. Shaw observed another curious fact, that the orbital cavities do not vary perceptibly in rachitic skulls from those of giants.

The pelvis becomes distorted, its antero-posterior diameter at the outlet is greatly diminished, while its transverse diameter is increased, which is said to differ from pelvic deformity due to osteo sarcosis, in which the lateral diameters are diminished, and the antero-posterior diameter increased.

Dr. Shaw's measurement of a number of rachitic pelves shows the average amount of deficiency in their diameter to be one quarter their normal diameters. His explanation is, that during intra uterine life, the upper half of the body grows more rapidly than the lower half, and after birth this process of development is reversed, and if rachitis appears this rapid growth of the lower half is suddenly checked, and the pelvis retains its infantile proportions in addition to deformity due to softening of its bony walls. Important changes take place in the shape of the thoracic walls, the blood vessels are sometimes changed in their courses. The position of the spinal column is altered and various abnormal conditions of the liver, heart, spleen, lungs, lymphatic glands etc. are often present.

Many of these changes are due to mechanical causes, and have little, if any, pathological bearing in the causation of the disease.

Of all the deviations from the structure of normal bone, none are more interesting and important than those of the minute anatomy of the bone tissue and adjacent parts.

The periosteum after being removed from the bone will be found vascular, somewhat thickened, and if it presents any of the evidences of inflammation, as *Mayer* says it does, they are not so well marked as in osteo-myelitis. It is the same writer who suggests that periostitis may be the proximate cause of rachitis. Underlying the periosteum is found a soft spongy substance of rather a deep purple color, and entangled in this can be seen recently formed ossific matter.

So long as pathologists disagree in the manner in which normal ossification is performed, it occurs to us that it will be impossible to determine exactly how a retrograde process can be followed up.

To make a general statement, it can be said, that in rachitic bone there is a reversal in the relative proportion of its organic and earthy constituents. A healthy bone contains one part organic and two parts inorganic matter. In a rachitic bone there are two of the former to one of the latter, by reason of the withdrawal of a portion of its inorganic elements.

*Rokitansky* says that this process may be affected in two ways, acting at the same time and independently of each other, either one of which may preponderate. In the first the bone is expanded, its canals and medullary spaces become enlarged and filled with a yellowish red or brown gelatinous substance; its blood vessels expand, become more numerous and fill with a dark colored grumous blood.

In the second, the mineral constituents disappear, leaving the bone of its original size and shape, but flexible and cartilaginous in structure.

What would be important to know, pathologists do not state in what tissue or structure of bone these processes begin; and what is more, I believe that most of the specimens examined have been dried or allowed to macerate for a considerable while, which allows time for further changes and wasting of its destructible material.

The hypothesis that lactic acid may dissolve the phosphate and carbonate of lime would seem to yield to the fact, that its presence in rachitic subjects is not constant. Often the vascular tissue of rachitic bone shows the presence of alkalies in excess. What seems curious and should have been mentioned while contrasting the two diseases, osteo-sarcosis and rachitis, Hintzman, of Vienna, concludes from experiments made, that the continuous administration of lactic acid to carniverous animals is capable of producing rachitis in the young, and osteo-sarcosis in the old ones; and that these two conditions are identical processes.

Vogel, Reindfleish, Humphry, Kolliker and Virchow are of the opinion that the preparatory processes go on normally, but become arrested just as solidification is about to commence. There appears no proof that the salts of lime are absent from the blood, and in most cases they are found so abundantly in the urine as to give it a muddy appearance, although some have failed to discover any of the phosphates in the urine in the cases they have examined, still there is no reason why the composition of the urine should not vary in rachitic persons as well as in healthy subjects.

Holmes when speaking of scrofula in bone, says the leading features of the pathological anatomy of strumous inflammation are the same as those of ostitis in general, and the only distinctive anatomical peculiarity consists in the nature of the exudation which chokes up the canals of the bone. Dr. Black concludes, after repeated analyses, that tuberculosis gives rise to a considerable increase of fat in the diseased bone, large diminution of the organic matrix, and an increase of soluble salts. The canals in the interior of scrofulous bone are enlarged and filled with a reddish jelly like material. The bone substance can be cut readily with a knife.

From pathological conclusions, it would seem, clear, that in rachitic bone, can be found all the attributes of inflammation, viz: increased vascularity, hyperæmia, morbid secretion, proliferation of the cell corpuscles in the connective tissue, destruction of normal tissue, etc.

Paget found a large amount of fat present, and believed rachitis was a process of fatty degeneration.

Von Bebra found in a radius of a rachitic child but 6 per cent. of fat. Stroymeyer is probably nearest correct, when he says the pathological lesions are identical with ostitis. Other pathologists are strongly inclined to favor this opinion, and nearly every writer on the subject declares that the bone changes are closely allied to inflammation, if indeed that is not precisely its true condition.

Those who recover from the disease after it has progressed to softening of the bones, remain more or less permanently distorted. The bones become unusually hard and brittle, some of the flat ones acquiring great thickness, which is well to remember when operating upon them. The deposition of ossific matter goes on beyond the limit of ordinary repair.

In flat bones which have become bent at an angle, by either superincumbent weight or muscular contraction while in a flexible state, it is found that at these points the vascular interior has become a solid bony mass, and probably for additional strength at this weak point, there appears a deposition of inorganic matter on its concave surface by a process not unlike that which takes place in the more simple forms of animal life, a process differing from that of repair of bone in the more complicated animals, following a general law that the reparative process of nature in the osseous system is in an inverse ratio to the complexity of the organism. Thus, nature repairs quicker and more permanently, the fractured shell of an oyster, turtle or lobster, than a broken bone in man.

We even observe in some of the lower animals a whole limb restored after having been torn from the trunk, and some of the *Radiata* if divided in pieces, a perfect animal may be developed from each subdivision.

In some the growth of bone is excessively exaggerated and knotty, growths spring from different parts of the skeleton which in many cases are unique in form and structure. Anchyloses of the joint surfaces and osseous union of all bones separated by a cartilaganous substance, is liable to occur. A case of this kind was reported in Ireland over two centuries ago, where the skeleton of a man, who died at the age of 61, was found to be composed of one continuous bone from the top of the head to the knees. This individual, it was said, had rachitis in early life.

In treating rachitis, practitioners have been guided very much by the theories they respectively entertain concerning the causation of the disease. On the hypothesis that the cause lies in a deficient supply of the lime salts; lime has been administered in all its forms, but without vary gratifying results. The advocates of the lactic acid theory, have likewise failed to cure their patients by prescribing with a view to antagonize this supposed chemical cause or lactic acid diathesis. As it is true that the blood in rickety subjects contains the salts of lime in normal proportions, so is it true that the tissue of rachitic bone sometimes responds to alkaline reagents, therefore it would seem that the fault must be not in the absence of one or the presence of the other, but in a misappropriation of the lime elements.

After a vast amount of experimentation in treating the disease upon hypothetical notions, the therapeutics have been singled down in the main to the identical substance they fed their children on the shores of the Baltic long ago, Cod Liver Oil. The emulsion is probably the most efficacious form to administer, not for the reason that it furnishes lime to the softened bones, but because it is less liable to produce nausea, is more easily retained in the stomach, possibly more readily digested and absorbed, and often no doubt acts kindly in correcting any irritable state of the alimentory canal, which is apt to coexist with the affection.

Some authors would treat the disease antiphlogistically with antimony, mercury, etc., in the first stage, on the ground that it is inflammatory in its nature and should be managed the same as inflammation in other tissues. I believe nearly all authors now concur in advising a supporting treatment from the beginning through all stages of the disease, with such modifications as each individual case would seem to require. During the stage of softening or deformation, physical exercise should be interdicted and absolute rest insisted upon, and through this period of repose, motion of all the joints ought to be made daily, lest, should be added anchylosis to deformity. Quinine, iron, etc., can be given judiciously in many cases and the patient placed upon nutritious diet, with milk in abundance. Surroundings should be made as pleasant and healthful as possible. Through the whole course of the disease out door air will be found invigorating to the patient.

With reference to the application of mechanical appliances to prevent the flexible bones from becoming permanently distorted, I shall say but little. General practitioners usually discredit their utility, while surgeons as often believe they can be made serviceable in most cases. Should they be resorted to great care must be taken, lest by pressure upon the soft parts they do more mischief than good.

